# A Case of Severe Herpes Zoster and Varicella Occurring Simultaneously in an Aged Patient

**Published:** 1926

**Authors:** R. E. Overton

**Affiliations:** Corsham, Wilts.


### A CASE OF SEVERE HERPES ZOSTER AND VARICELLA OCCURRING SIMULTANEOUSLY IN AN AGED PATIENT.

BY

R. E. Overton, M.R.C.S., L.R.C.P.

(*Corsham, Wilts.*)

---

The patient was a remarkably active old gentleman of 93 years and 6 months. He had enjoyed excellent health all his life, and at the onset of his illness was driving out each day and taking a lively and controlling interest in his affairs. The only physical defect was an ankylosis of his left hip joint and some three inches of shortening in the left leg following fracture of the neck of the femur thirty years ago.

On April 18th, 1926, I was consulted, as Mr. X. had then been complaining for three days of—

(1) Slight sore throat.

(2) Stabbing pains in the right side of the neck and jaw and over the right shoulder. The pain was instantaneous, and seemed to " dart right through his head."

(3) Insomnia owing to the pain—the latter being worse at night.

The patient looked ill. He was fully dressed and unwilling to submit to a proper examination. Such of the skin as could be inspected appeared normal. There was no hyperæsthesia, and at the time of the consultation the patient was quite comfortable and free from pain.

Phenazone gr. x was prescribed, to be repeated every six hours. The patient had a fairly comfortable night.

April 19th, 1926. The following day an extensive rash of herpes zoster was present over the whole area of the third and fourth cervical nerves on the right side. Many of the vesicles contained a dark blood-stained fluid. A great number had been scratched and were bleeding. The vesicular area was surrounded by a small margin of erythema. The temperature was normal, but the pain continued in severity and frequency.

An ointment containing 3 per cent. yellow mercuric oxide was applied on lint over the whole area.

April 20th, 1926. The day after the rash was at its maximum. Large areas had become infected, and there was considerable sloughing of the skin. The region which had been pressed on by the back collar-stud was the worst, and here quite a deep slough was present.

April 21st, 1926. On the third day of the rash the dressing had to be discontinued owing to a mercurial dermatitis. It was replaced by calamine lotion and starch poultices in the area of the beard.

April 22nd, 1926. Lymphatic enlargement in the right axilla was considerable and painful. There was very marked œdema of the right arm and dorsum of the hand.

April 23rd, 1926. The patient's general condition gave rise to some alarm. Pain, lack of sleep and the disturbances necessitated by the dressings had fatigued him considerably. In view of his advanced age and his own inclinations it was considered inadvisable to insist on his taking to his bed.

April 24th, 1926. On the fifth day after the appearance of the herpes the patient developed a generalised varicella. The vesicles extended over the whole body, most marked on the back and abdomen. (About two hundred vesicles were present altogether.) A few vesicles were present on the face and limbs, and about a dozen on the scalp. This rash followed the course of a perfectly typical chicken-pox, passing through the papular, vesicular and scab stages. In ten days from the commencement of the varicella all evidence of it had disappeared.

May 4th, 1926. By this time the herpetic area had almost completely healed. In this same area, however, severe attacks of shooting pains were still a constant feature ; itching and the sensation of " small animals crawling about " were causing a great deal of discomfort. The enlargement of the axillary lymphatics subsided with the sepsis. Sleep could only be obtained artificially, gr. xx each of chloral and bromide being given alternately at night with gr. v of barbitone.

The general disturbance of the chicken-pox appeared to be much greater than that of the herpes, so that on May 5th the patient had become so feeble that he was unable to leave his bed. He then for the first time became completely helpless and despondent. The herpetic lesions had quite healed, but in that area the loss of sensation was almost complete ; in only very few of the least involved areas was the patient able to feel sharp stabs with a pin. Discrimination between hot and cold was entirely absent.

May 15th, 1926. After a period of ten days, during which the patient was perfectly helpless and bedridden, he began slowly to improve.

June 17th, 1926. He now drives out again every day, and appears to be as well both physically and mentally as he was before the onset of the disease.

Mr. X. tells me—and his memory is extremely good—that he has never had either of these diseases before.

It is over thirty years since the first account was published of cases in which herpes zoster was associated with varicella. Reviews of the subject have been published from time to time, and of late interest has been revived by reports of cases in this country, in France and elsewhere. A perusal of these records (as abstracted in recent volumes of the *Medical Annual*) shows that there are three kinds of reported association ; chicken-pox may arise after contact with herpes in another person, herpes may similarly arise after contact with chicken-pox, or the eruption of varicella may follow that of herpes after an interval of a few days, in the same individual.

It appears, also, that three explanations of this association have been advanced. First, it is suggested that the eruption described as varicella is really that of herpes zoster generalisatus. To the present instance this hypothesis does not seem applicable. The two eruptions each ran their course typically and separately. That of herpes was strictly limited to the original area, and so were the nervous phenomena which lingered after its subsidence. The varicellar rash, on the other hand, was scattered after its normal fashion over the trunk, and its component elements passed through the stages customary in varicella.

Second, it is sought to explain the association away as a mere coincidence. Both for and against this hypothesis a great many arguments have been marshalled, and a single case can scarcely move the balance of opinion in either direction. During the period when this patient's case was under observation about a dozen examples of chicken-pox, on the one hand and a similar number of cases of herpes, on the other, were noted in the same district. Was this a coincidence, or was it a further piece of evidence in support of the third hypothesis ?

This seeks to prove an identity of causation for the two diseases ; no easy task, since we are so far without clear knowledge of the virus of either. Much has been made, on both sides, of arguments based on the immunity said to be conferred by one disease against the other. In this instance the patient was immune against neither. The fact that he had escaped immunisation until an advanced age might serve to explain the severity of the infection when at last it did attack him.

